# Classification of Gait Type Based on Deep Learning Using Various Sensors with Smart Insole

**DOI:** 10.3390/s19081757

**Published:** 2019-04-12

**Authors:** Sung-Sin Lee, Sang Tae Choi, Sang-Il Choi

**Affiliations:** 1 Department of Data Science, Dankook University, Yongin 16890, Korea; leesungsin@gmail.com; 2 Department of Internal Medicine, Chung-Ang University, Seoul 06984, Korea; 3 Department of Computer Science and Engineering, Dankook University, Yongin 16890, Korea

**Keywords:** gait type classification, deep learning, feature extraction, sensor array, smart insole

## Abstract

In this paper, we proposed a gait type classification method based on deep learning using a smart insole with various sensor arrays. We measured gait data using a pressure sensor array, an acceleration sensor array, and a gyro sensor array built into a smart insole. Features of gait pattern were then extracted using a deep convolution neural network (DCNN). In order to accomplish this, measurement data of continuous gait cycle were divided into unit steps. Pre-processing of data were then performed to remove noise followed by data normalization. A feature map was then extracted by constructing an independent DCNN for data obtained from each sensor array. Each of the feature maps was then combined to form a fully connected network for gait type classification. Experimental results for seven types of gait (walking, fast walking, running, stair climbing, stair descending, hill climbing, and hill descending) showed that the proposed method provided a high classification rate of more than 90%.

## 1. Introduction

Gait is one of the most common behaviors of everyday life. If there is a problem with one’s gait patterns, it may indicate the presence of not only musculoskeletal disorders such as deformation of the joint [1], but also mental diseases such as intellectual disability [2], dementia [3] and depression [4]. In addition, accurate measurement of the amount of exercise during daily life is one of the most important factors for performing and evaluating exercise therapy and rehabilitation of patients. Therefore, many studies on gait have been carried out. Methods to classify types of gait in particular have received much attention in various health care fields [5,6,7] including the field of medical diagnosis [8].

Various methods have been proposed to automatically classify gait types based on data obtained by measuring gait pattern. In a previous study [9], a method to distinguish between straight and curved walking using a gyroscope sensor and a pressure sensor has been proposed. In another method [10], the angle at which the ankle joint moves on a treadmill is calculated using a pressure sensor and an inertia measurement unit (IMU) sensor. Stance, heel-off, swing, and heel-strike are calculated and four parts of the gait cycle (stance, heel-off, swing, and heel-strike) are then found based on a certain threshold value [10]. However, since the gait cycle is determined based on the amount of angular change of the ankle joint and the threshold value set by the user which is the criterion used for determining angular change amount, it is limited in that it only allows for the identification of gait cycles when certain constraints are set. In another study [11], various kinds of sensors such as acceleration, gyroscope, and humidity meter sensors are attached to eight body parts and behavior data are collected from settings of home, bus, restaurant, and library. Types of gait and behavior are then identified using a decision tree and artificial neural network. However, this method has disadvantages associated with the need to attach too many sensors to the body to collect data, as well as the high complexity of the model defined to classify the data. In [12], a method has been proposed to calculate the number of steps and the distance actually moved after collecting gait data for walking, sideways, and running using an acceleration sensor.

Various gait analysis methods using a hidden Markov model (HMM) have also been introduced. In [13], gait phases are classified using HMM with an IMU sensor attached to legs and switches attached to the sole. In [14] and [15], human identification and gait type classification have been performed using HMM based on IMU sensor data, respectively.

Gait analysis methods using machine learning techniques have been proposed. In [16], kinematic data are measured by using IMU sensor attached to thigh and knee angle sensor and gait phases are classified using machine learning techniques. In [17,18], spatial-temporal gait features such as stride length, cadence, stance time, and double support time were obtained using techniques such as pressure-sensitive GaitRite mats or foot switches. Walking patterns of patients with Parkinson’s Disease Stroke are then analyzed by using SVM, random forest [17], or mixture-model [18]. In [19], the trajectories of rotational angle and global velocity of selected body joint are obtained using two depth cameras and pathological gait was classified by using k-nearest neighbor classifier. A method has been proposed to distinguish between gait patterns in normal and Parkinson’s Disease patients using artificial neural networks [20]. However, 37 reflective markers are needed to be attached to the skin of the experimenter and data are collected using six infrared cameras which can restrict the use environment. In [21], a method for recognizing people has been proposed using a convolutional neural network (CNN) [22] for optical flows from video images. In [23], a 1D convolutional neural network is used to classify three axes of acceleration (*x*, *y*, *z* axis) data acquired using a smartphone into three walking patterns (walking, running, standing). Since this method measures gait with a smartphone in hand or in one’s pocket, the movement of the hand during walking or the movement due to squeezing of the pocket is reflected in the gait data, making it difficult to accurately measure gait pattern. In addition, there are limits to how input data are defined during the pre-processing of data, resulting in changes in classification performance.

Meanwhile, as sensor modules have become smaller and low-power sensor technologies have been developed, various wearable devices such as smart watches, sports bands, and smart insoles have been developed. Since the use of wearable sensors has less environmental limitations for data collection, it is relatively easy to collect data in everyday life. This has advantages of reduced data storage and processing burden because it is smaller in capacity compared to the use of video data such as optical flow and heat map. Among gait analysis methods using smart insole, the method in [24] can classify gait types of elderly people using pressure, acceleration, and gyro data of smart insoles by applying a decision tree based on various conditions. However, this method has a problem of incorrectly classifying some types of gait because it classifies based on conditions set by the experimenter. In [25], pressure sensor data for walking are classified by kernel based principal component analysis (KPCA) and support vector machine (SVM) based on walking pattern. However, this method reflects variations that are not helpful for walking classification when projecting data on axes orthogonal to each other while preserving the maximum variance of data. Thus, it is unsuitable for classifying data having various classes or variations in gait pattern. In [26], seven types of gait were classified using discriminant analysis [27] with pressure data of a smart insole [26]. However, it is necessary to measure multiple steps in order to obtain reliable classification performance. In addition, it could not reflect various characteristics of gait because it only uses one pressure sensor.

In this paper, we proposed a method to classify gait types based on deep learning using gait data measured by various types of sensors installed in a smart insole. For data collection, we used pressure, acceleration, and gyro sensors of ’FootLogger’, a commercial smart insole [28]. We constructed a deep neural network for data classification based on deep convolutional neural network (DCNN) [22]. The proposed method consists of a pre-processing part for segmenting the continuous gait data into unit steps followed by the data normalization and a classification part for determining the type of gait by extracting features using deep neural networks. In the pre-processing stage, continuous gait data are precisely segmented by eliminating noise generated during the sensing process based on characteristics of the swing phase during the gait cycle. In the classification stage, we constructed multiple DCNNs for each kind of sensor mounted on the smart insole and then extracted feature maps for the data obtained from each sensor. Feature maps were then combined and a fully connected network was constructed to determine the type of gait. Experimental results of seven types of gait data for 14 adults aged 20 to 30 years old showed excellent classification performance of the proposed method.

The proposed method can be applied to data evaluation important for exercise therapy and rehabilitation patients with various diseases such as arthritis, osteoarthritis, rheumatoid arthritis, and neurologic disorders related to stroke and Parkinson’s disease. Since sarcopenia is closely related to the deterioration of these diseases, increasing muscle mass of the lower extremity is very important in the treatment of these patients. Muscle mass can vary depending on how muscles are used throughout the day. In addition, accurately assessing patient ’s activity (or exercise) pattern and adjusting the manner and amount of activity in daily life accordingly can be of great help in exercise therapy and rehabilitation of these patients.

This paper is organized as follows. Section 2 presents the smart insole used to acquire gait data as well as the pre-processing of measurement data. Section 3 describes the design of deep neural networks for classification. Section 4 presents experimental results of gait type classification. Section 5 then concludes this paper.

## 2. Data Acquisition and Pre-Processing

### 2.1. Data Acquisition

**Figure 1:**
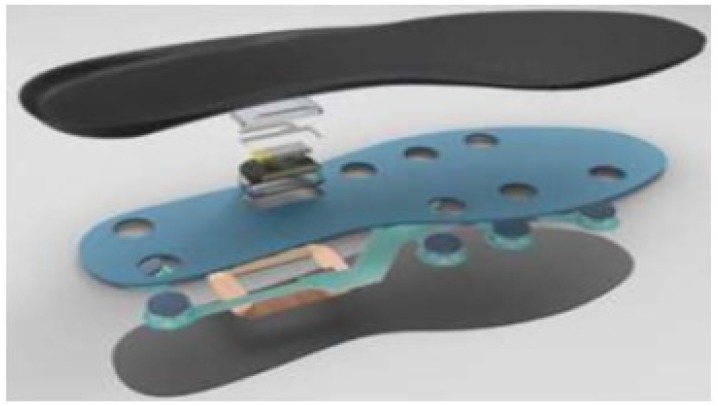
Sensor structure of smart insole ’FootLogger’.

In this paper, ‘FootLogger’ (Figure 1), a commercial smart insole [28] was used to collect gait data. The ‘FootLogger’ incorporates a pressure sensor array consisting of eight pressure sensors, a three-axis acceleration sensor array, and a three-axis gyro sensor array. These sensors mounted on both insoles can measure data at a sampling rate of 100 Hz. In the case of a pressure sensor, the value is measured as 0, 1, or 2 depending on the intensity of the pressure. A value of 0 indicates that there is no pressure, that is, a swing phase (feet away from the ground). Values of 1 and 2 indicate a stance phase (the pressure in the state when the foot is touching the ground). These measured data are transmitted to the database server through a bluetooth applicaton using an Android smartphone.

### 2.2. Unit Step Segmentation

**Figure 2:**
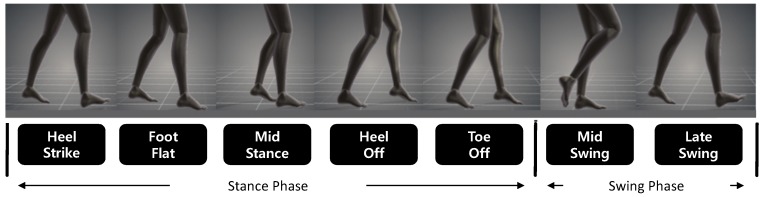
Gait cycle.

A gait cycle [29] is the period from when a foot touches the ground to when it falls from the ground and reaches the ground again. As shown in Figure 2, the gait cycle consists of seven stages (‘heel strike’, ‘foot flat’, ‘mid stance’, ‘heel off’, ‘toe off’, ‘mid swing’, and ‘late swing’). The ‘heel strike’ stage represents the beginning of the gait cycle, where the heel of the first moving foot touches the ground, while the sole of the moving foot touches the ground at the ‘foot flat’ stage. In the ‘mid stance’ stage, the leg of the first moving foot is perpendicular to the ground and momentarily stopped, while the other leg moves forward. The ‘heel off’ stage refers to the time when the heel begins to move away from the ground, and the ‘toe off’ is the step where the anterior toe of the first moving foot leaves the ground while the sole of the other foot touches the ground. The ‘mid swing’ is the step forward with the foot off the ground. Finally, the ‘late swing’ refers to the stage immediately before the next gait cycle begins. When viewed from one foot, ‘heel strike’, ‘foot flat’, ‘mid stance’, ‘heel off’, and ‘toe off’ stages are defined as the ‘stance phase where the foot touches the ground. The ‘mid swing’ and ‘late swing’ stages that are separated from the ground, are defined as the ‘swing phase’.

**Figure 3:**
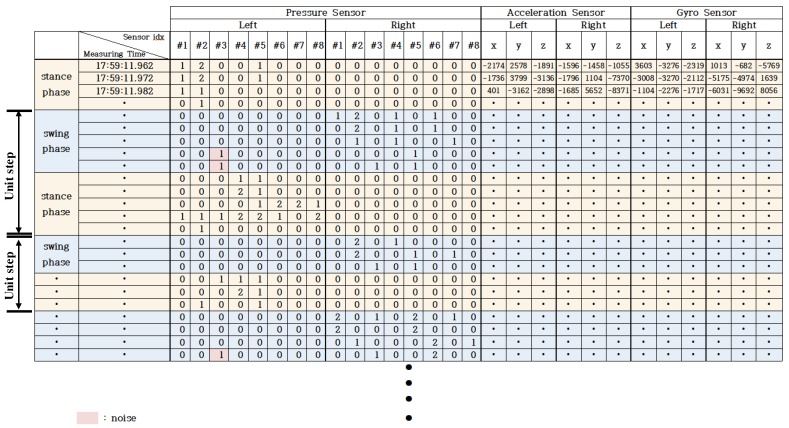
Example of gait data measured using ‘FootLogger’.

In order to continuously segment measured data into unit steps during gait, we divided unit step length based on the stance phase and the swing phase of the gait cycle [30]. Figure 3 shows an example of gait data measured using the ’FootLogger’. In the data of the pressure sensor array shown in Figure 3, it can be seen that the swing phase is where values of all pressure sensors of the array become zero successively, and the stance phase is where values of some pressure sensors are measured as 1 or 2. The swing phase alternates between the left foot and the right foot during gait. In this paper, unit data sample corresponded to one step from the starting point of the swing phase to the end point of the stance phase for the left foot.

Unit step data samples are stored in the form of a matrix for each of pressure sensor arrays, acceleration sensor array, and gyro sensor array. Column and row of the matrix are indices of each sensor at the time of measurement, respectively. As a result, in the case of a pressure sensor array consisting of eight sensors per foot, stored data constitute a matrix with 16 columns while the data for the acceleration sensor array and the gyro sensor array are stored in the form of a matrix with six columns.

### 2.3. Noise Reduction

In the ’swing phase’ of the gait cycle, since one foot is separated from the ground, all eight pressure sensors for that foot should have a value of zero. However, when measuring the data using the ’FootLogger’, a non-zero noise value was occasionally measured in a specific sensor even though it was in the swing phase. This was due to various factors, including potential difference between sensors mounted on the insole and heat generation (specifically, in the case of the product used in this experiment, the value of the third sensor was sometimes measured as one in the swing phase).

In the proposed method, since the unit step is divided based on the swing phase, this noise can cause an error that mistakenly judges that one swing phase has occurred twice which can deteriorate the classification performance of the proposed method.

**Figure 4:**
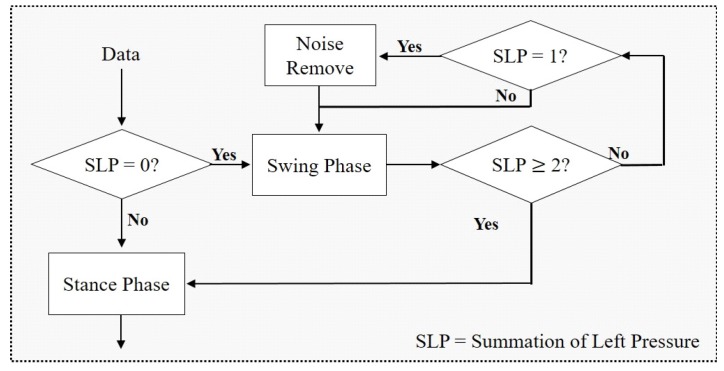
Flow of noise reduction in the swing phase.

Figure 4 shows the process of removing noise in the swing phase. First, the starting point of the ’swing phase’ is defined as the point at which the sum of all eight sensor values of the left foot pressure sensor array becomes zero [30]. The starting point of the stance phase following the swing phase is defined as the point at which a non-zero value is measured by two or more of the eight pressure sensors. In the case that the sum of the sensor arrays between the starting point of the swing phase and the starting point of the stance phase is not 0 but 1, the pressure sensor value at that point is determined as noise. Thus, the sensor value is changed to 0.

### 2.4. Unit Step Data Sample Normalization

Even in the same person, walking speed can vary during data measurement or when data are measured at different times. As such, variations in walking speed not related to the type of gait can interfere with the extraction of gait characteristics for the purpose of distinguishing the type of gait.

**Figure 5:**
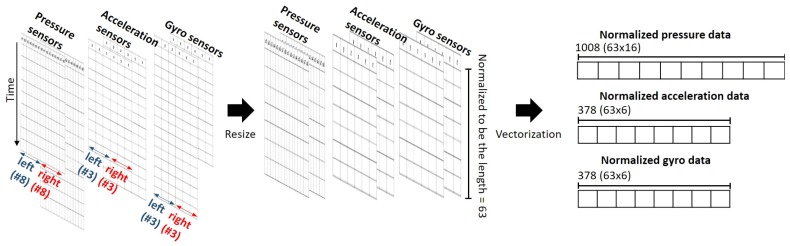
Raw data and the normalized data for the unit step.

To extract features that are less sensitive to environments when measuring data, all unit steps were resized based on the time (*t*) of the shortest unit step (in this paper, *t* = 63) so that lengths for all steps were normalized [26]. As a result, measurements for normalized unit steps of the pressure sensor array, the acceleration sensor array, and the gyro sensor array were converted into arrays of 63 × 16, 63 × 6, and 63 × 6, respectively, and then stored as vectors (**x**) of 1008 × 1, 378 × 1, 378 × 1, respectively, using a lexicographic ordering operator. Figure 5 shows raw data as well as normalized data for the unit step.

## 3. Design of Deep Neural Network for Classification

### 3.1. Deep Neural Network Architecture

Gait data measured using the ‘FootLogger’ are time series data measuring sensor values at intervals of 0.01 s. Since human gait is a continuous operation, the correlation between sensor measurements at each time point is relatively large. Therefore, in this paper, we designed a deep neural network, DCNN (Deep Convolutional Neural Network) [31], which can utilize data correlation and classify gait types based on various sensor measurement values.

**Figure 6:**
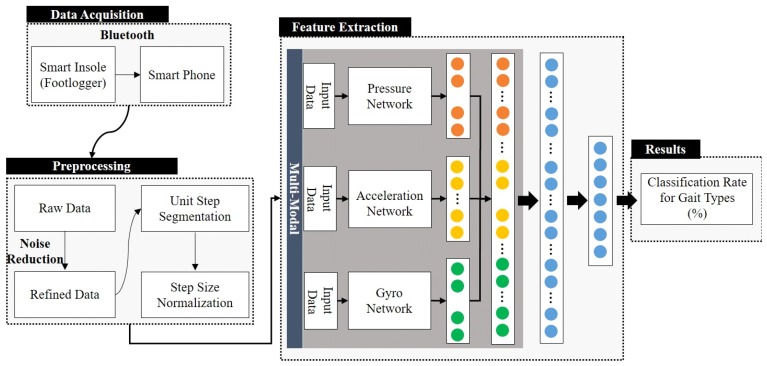
Overall structure of the proposed method to classify gait types (multi-modal DCNN).

In general, a convolution-based artificial neural network consists of a feature extractor and a fully connected network. The feature extractor contains three types of layers: filter layer, nonlinear activation function layer, and feature pooling layer [22]. Figure 6 shows the overall structure of the proposed gait type classification method. As shown in Figure 6, a network is constructed for each of the pressure sensor array, the acceleration sensor array, and the gyro sensor array data. It was learned independently in order to extract feature maps for individual sensor arrays.

### 3.2. Network Configuration

**Figure 7:**
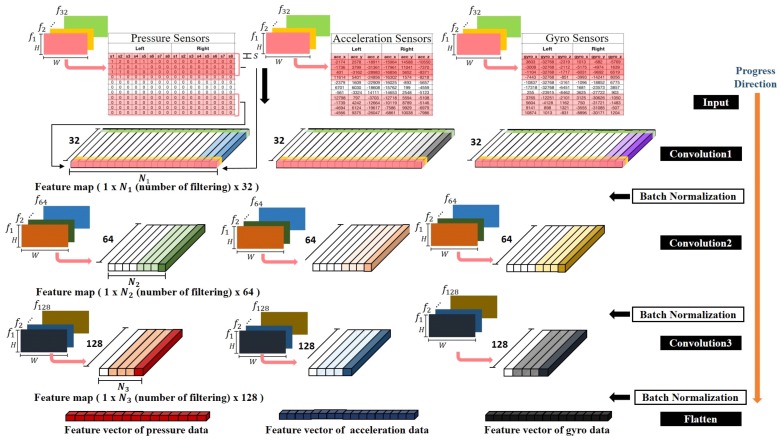
Structure of an individual DCNN for each sensor array.

- Input Data Format
  The DCNN receives data in the form of a two-dimensional array and performs a convolution operation with various filters in the convolution layer. In this paper, measured data for each sensor array are normalized to t×W size through the pre-processing process mentioned in Section 2. These normalized data are used as input of DCNN. *W* is the number of sensors in the sensor array and *t* is 63. For pressure sensor arrays, acceleration sensor arrays, and gyro sensor arrays, *W* values are 16, 3, and 3, respectively. In order to determine how many steps are necessary to extract gait characteristics for the purpose of distinguishing gait types, classification experiments are performed using data samples consisting of one step. The number of steps included in a data sample is then continuously increased to perform classification experiments at each increment. For example, if one gait data sample is defined as *k* steps, the input data size of DCNN becomes (t·k)×W.

- Convolutional Layer
  The DCNN used in the proposed method includes three convolution layers. Figure 7 shows the structure of an individual DCNN for each sensor array. Each layer contains filters at the corresponding feature level. For convolution operation at each layer, the following three hyper-parameters should be determined: the number of filters to use (*f*), the size of filters (W×H), and stride (*s*). In the first convolution layer, a total of 32 filters are used and the size of the filter is set to be $W_{1}\times 20$ (*H*). The filtering stride is set differently according to the number of steps (*k*) included in the input data of DCNN (s=1 for k=1,2; s=2 for k=3,4,5). The second and third convolution layers use 64 types and 128 types of filters, respectively. The size of the filter is differently set according to the size of the output signal from the previous layer. It is set to be 1 × 20 and 1 × 20 for the second and the third convolution layers, respectively (Figure 7). The 1D convolution operation between the filter and the input data outputs a single scalar value. Operations of all filters at a certain position of the input data generate a feature vector. If convolution operations are performed while moving the position of the filter by *s*, a feature map of $N_{l}$, l=1,2,3, (the number of filtering) × 32 (the number of filters) is then generated.

The DCNN was trained using a back-propagation algorithm [32]. In the learning process, when the number of layers of the neural network increases, differential values of weights in the back propagation of the error are close to zero (‘vanishing gradient phenomenon’ that occurs when the gradient can not be transmitted. In order to prevent this phenomenon, we used ReLU (Rectifier Linear Unit) [33] as an activation function. To prevent an internal covariance shift phenomenon in which the distribution of input data continuously changes due to the nonlinear activation function used in each layer of the neural network, batch normalization [34] was performed after batch activation function was applied in order to enhance the stability of learning. The batch size was set to be 32. The DCNN independently learned the feature map of each sensor type for each sensor array output.

**Figure 8:**
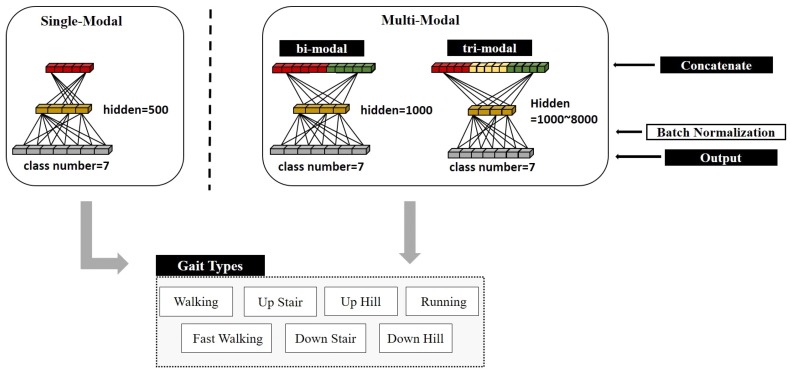
Structure of a fully connected network that determines the type of gait using the single- and multi-modal DCNN feature vector as input.

- Fully Connected Network and Output
  Each feature map is transformed into a DCNN feature vector (flattening), which is used as an input to a fully connected network. The fully connected network consists of two layers (except for the output layer). In order to avoid over-fitting problems and improve regularization performance, we randomized nodes in layers and dropped the remaining nodes before transferring values to the output layer. This is called ‘dropout’ [35] (in this paper, the experiment was conducted by changing the dropout ratio from 0.5 to 0.7).
  The output layer consists of as many nodes as the number of classes that are there to classify. At each node, the sum of weights for nodes of the previous layer is applied to the softmax function in order to calculate the final output value. In this paper, we constructed a single-modal DCNN and a multi-modal DCNN depending on the number of sensor arrays used. In the single-modal DCNN, the feature vector extracted from one kind of sensor array was used as the input of the fully connected network. In multi-modal DCNN, feature vectors of two or more sensor arrays were connected together and used as input into the fully connected network. Figure 8 shows structure of the fully connected network that determines the type of gait using single- and multi-modal DCNN feature vector as input.

## 4. Experimental Results

### 4.1. Data Measurement

Gait data were collected from 14 adults in their 20 s and 30 s, measuring seven types of gait: walking, fast walking, running, stair climbing, stair descending, hill climbing, and hill descending. For walking, fast walking, and running, data were measured for three minutes. For hill climbing, hill descending, stair climbing, and stair descending, data were measured from the starting point to the arrival point, regardless of the time needed (around one minute to two minutes). Gait measurements of each person were divided into unit steps and normalized through data pre-processing described in Section 2. Information on gait data used in the experiment is summarized in Table 1.

**Figure 9:**
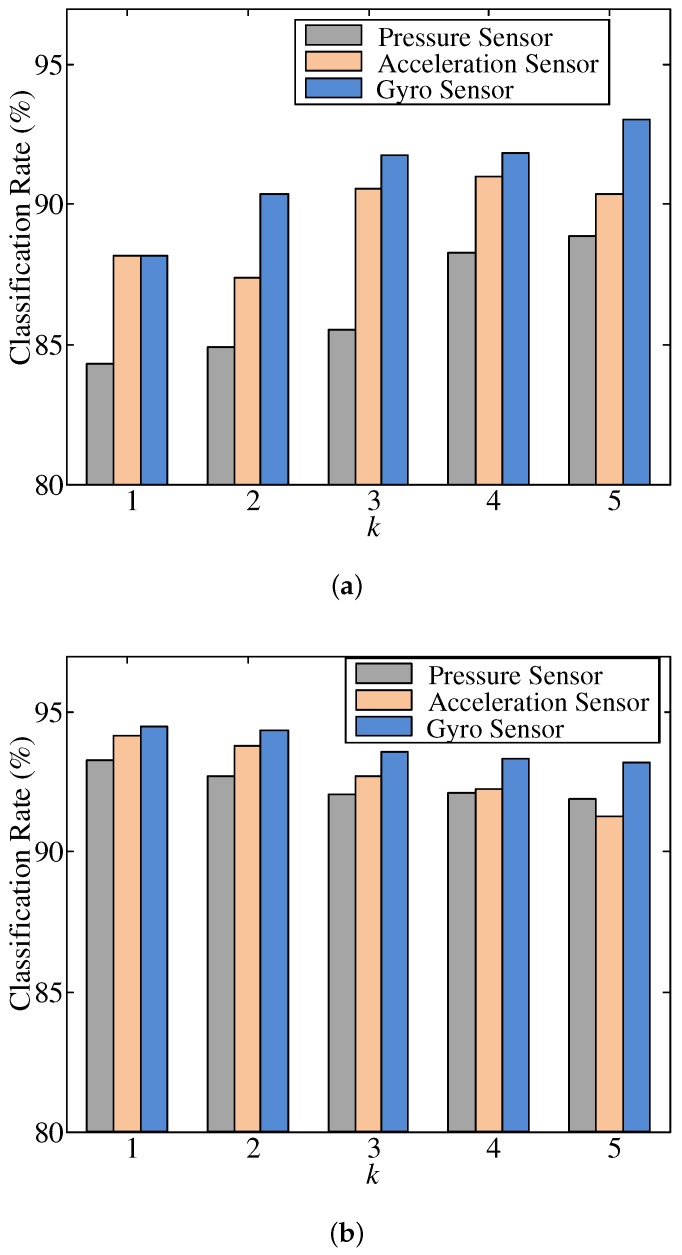
Classification rates of single-modal DCNNs for various *k*. (**a**) randomly selected 1000 training data samples and 1000 test data samples; (**b**) 7-fold cross validation

**Figure 10:**
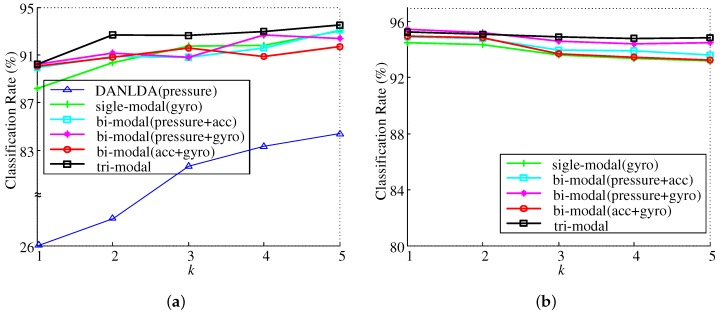
Classification rates of multi-modal DCNNs for various *k*. (**a**) randomly selected 1000 training data samples and 1000 test data samples; (**b**) 7-fold cross validation.

In order to evaluate the classification performance, data sets for training and testing were constructed by randomly selecting 1000 data samples from all data. To increase statistical confidence, the above procedure was repeated 20 times and the average classification rate was calculated (Figure 9a and Figure 10a). We performed an additional *K*-fold cross validation experiment (Figure 9b and Figure 10b). In *K*-fold cross validation, total samples were randomly partitioned into *K* equal sized subsets. Of *K* subsets, a single subset was retained as a test set for validating the model while the remaining K−1 subsets were used as a training set. The cross validation process was then repeated *K* times. Each subset was used exactly once as a test set. Since *K* was usually set to the number of classes, we performed a 7-fold cross validation in our experiment. To increase statistical confidence, the above procedure was also repeated 10 times and the average classification rate was calculated.

In order to determine the number of steps required to obtain information that could distinguish the type of gait, we investigated the classification rate by gait samples composed of steps from one step (k=1) to five steps (k=5). The training data samples were normalized to have zero mean and unit variance, and the test data samples were also normalized using the means and variances of the training data samples. Table 2 shows total number of gait data samples according to the value of *k* and the number of training data samples and test samples in 7-fold cross validation experiments for each *k*.

### 4.2. Classification Performance

For classification experiments, Matlab 2018a (Mathworks, Natick, MA, USA) was used in Windows operating system environment and Keras 2.1.5 (https://keras.io/) was used for the DCNN related library. ’Categorical Cross Entropy’ was used as a loss function to calculate the difference between the output value and the correct label value. Adaptive momentum optimizer [36] was used to optimize the weight of the network. The learning rate of the adapt optimizer was set to be 0.0001.

The classification performance was evaluated by a single-modal DCNN experiment using a feature map extracted from one kind of sensor array data as well as a multi-modal DCNN experiment in which a network was learned together with data from two or more sensor arrays. Figure 9 shows results of classification of gait type using the single-modal DCNN, which was trained independently for each kind of sensor array.

In Figure 9, among features maps of the three kinds of sensors arrays, the performances of acceleration sensor and gyro sensor are shown to be slightly higher than that of the pressure sensor array. It can be considered that the gait pattern produced by information of foot motion is useful for distinguishing the type of gait.

Figure 9a shows results of using 1000 training samples and 1000 test samples for all *k*. As seen in Figure 9a, when the number of steps included in gait data sample increased, the classification rate increased. However, starting from three steps or more, the increase in the classification rate was either small (for the pressure sensor and gyro sensor) or otherwise decreased (for the acceleration sensor). This means that we can extract more quality features for gait classification by constructing data samples with more steps and using those samples in feature extraction. However, if more than a certain number of steps are gathered, it appears that additional useful information is not actually obtained or that unnecessary information gathered can hinder effective feature extraction.

On the other hand, as shown in Figure 9b, since the number of training data was the largest at k=1, the highest classification rate was shown at k=1. This means that, even if data are measured over a short period, better features can be extracted if a sufficient number of data samples are obtained.

Figure 10 shows experimental results of the single-modal DCNN and various multi-modal DCNNs. In Figure 10, ‘bi-modal’ means the result of using two kinds of sensor arrays together, and ‘tri-modal’ means that three kinds of sensor arrays are used together. In order to clearly show the effectiveness of the proposed method, we compared it with DANLDA, the most current method [26] using a smart insole. Since the method of [26] uses only a pressure sensor, we first compared the single-modal DCNN (gyro) with DANLDA. In the 7-fold cross validation experiment, when *k* is 3 or less than 3, there is no null space because the number of training data samples is larger than the dimension of the data, and thus we show only the results of single-modal and various multi-modal DCNNs in Figure 10b. As shown in Figure 10a, the single-modal DCNN had higher classification performance, ranging from 8.49% (*k* = 5) up to 62.06% (*k* = 1) than DANLDA. In particular, while DANLDA can not effectively extract features for gait classification from gait samples with one step, the proposed DCNN can extract information about the type of gait even if only one step is measured. Consequently, classification rates are as high as 84.32% to 88.17% depending on the kind of sensor array being employed (Figure 9).

In Figure 10, we can see that the classification performance of the multi-modal DCNN is better than that of the single-modal DCNN. In addition, using three types of sensor arrays (tri-modal) can lead to a higher classification performance than using just two types of sensors (bi-modal). In Figure 10a, the classification rate of the multi-modal DCNN increased when the number of steps included in one gait sample was increased, similar to the case with single-modal DCNN. On the other hand, the classification performance of the multi-modal DCNN was more saturated at fewer steps (smaller *k*) than that of the single modal DCNN. This is due to synergy when combining various characteristics of different kinds of sensors.

## 5. Discussion

A large number of data samples are required for learning of a deep neural network. In our DCNN network for single-modal data, 32, 64, and 128 filters of 20 × *W* (*W* was set differently depending on the kind of modal) were used in each of the three convolution layers, respectively. The feature map of the third convolution layer was flattened and used as input of the fully connected layer which produced the final result. Since the number of data samples were insufficient compared to the number of parameters to be tuned, we constructed the network with as few convolution layers (three layers) as possible and set the initial value of parameters using the ‘Xavier uniform’ method [37] provided by the Keras framework widely used in general deep learning research. In addition, the batch normalization method as one of deep neural network learning techniques used in this paper, not only prevents the internal covariance shift phenomenon, but also suppresses ‘over fitting’.

The proposed method of analyzing the pattern of gait by extracting features from wearable sensor data using a deep learning network can be used in various fields as well as classifying types of gait. Since the pattern of gait is an innate characteristic of an individual, it can be used as a biometric for personal identification purposes. In particular, since the proposed method uses a wearable sensor equipment (smart insole), the data measurement environment is less restrictive than the video-based gait analysis method previously used for identification purposes. In addition, based on information about the normal gait pattern of an individual, it can be used for early diagnosis of diseases requiring long-term follow-up such as Knee osteoarthritis or Parkinson’s Disease.

## 6. Conclusions

In this paper, we proposed a method for classifying various types of gait by extracting features based on a deep convolution neural network from measured data using various kinds of sensor arrays mounted on a smart insole. Seven types of gait data were measured using a pressure sensor array, an acceleration sensor array, and a gyro sensor array built into the ‘FootLogger’, a commercial smart insole.

The proposed method consists of pre-processing of data and DCNN-based data classification. In the pre-processing stage, we removed noise of the data and normalized unit steps in order to eliminate several variations of gait that could interfere with gait type classification. In the classification stage, feature maps, each of which is independently learned using DCNN for each kind of sensor array, are extracted from the pre-processed data. We combined these feature maps and used them as an input of a fully connected network to determine the type of gait. As a result of the classification experiment using data of 14 adult walkers, the classification performance of the multi-modal DCNN using feature maps of two or more kinds of sensor arrays was superior to that of the single-modal DCNN using a feature map extracted from one kind of sensor array. The classification performance was also improved when gait sample was constructed by gathering data of several steps.

In the proposed method, features of multi-modal data are obtained by concatenating features extracted using deep learning network for each modal data. However, since the degree of contribution of each modal to the classification of the gait type will be different from each other, in order to utilize multi-modal data more effectively, it is necessary to evaluate the importance of features of individual modal and analyze the complementary relationship between features of each modal. For this, we plan to measure additional data for people of different ages in the future. In particular, recent studies on multi-modal deep learning network have been introduced. Based on such results, we plan to analyze the gait pattern more effectively by using multi-modal data. We will also perform studies to increase synergy between each approach through a more in-depth comparative analysis of existing methods for gait type classification including HMM based methods.

## Figures and Tables

**Table 1:** Information on gait measurement.

Gait Type	Number of Steps	Measuring Time	Average Time per Ten Steps
Walking	2294	3 min	9.1
Fast walking	2714	3 min	10.8
Running	3642	3 min	14.5
Stair climbing	747	1 min	8.9
Stair descending	971	1 min	11.6
Hill climbing	1577	2 min	9.4
Hill descending	1586	2 min	9.4

**Table 2:** The total number of gait data samples according to the value of *k* and the number of training and test samples in each iteration of 7-fold cross validation

*k*	Total Number of Gait Samples	Number of Training Samples	Number of Test Samples
1	13,531	11,598	1933
2	6742	5778	964
3	4476	3836	640
4	3347	2868	479
5	2671	2289	382
